# Emotional and behavioral problems, quality of life and metabolic control in NTBC-treated Tyrosinemia type 1 patients

**DOI:** 10.1186/s13023-019-1259-2

**Published:** 2019-12-04

**Authors:** Kimber van Vliet, Willem G. van Ginkel, Rianne Jahja, Anne Daly, Anita MacDonald, Corinne De Laet, Roshni Vara, Yusof Rahman, David Cassiman, Francois Eyskens, Corrie Timmer, Nicky Mumford, Jörgen Bierau, Peter M. van Hasselt, Paul Gissen, Philippe J. Goyens, Patrick J. McKiernan, Gisela Wilcox, Andrew A. M. Morris, Elisabeth A. Jameson, Stephan C. J. Huijbregts, Francjan J. van Spronsen

**Affiliations:** 1 Beatrix Children’s Hospital, Groningen, Division of Metabolic Diseases, University of Groningen, University Medical Center Groningen, CA33, PO box 30.001, 9700 RB Groningen, Netherlands; 2 Birmingham Children’s Hospital, Birmingham, UK; 3 Hôpital Universitaire des Enfants Reine Fabiola, Université Libre de Bruxelles, Brussels, Belgium; 4 Evelina London Children’s Hospital, London, UK; 5 Guy’s and St. Thomas’ Hospital, London, UK; 6 University Hospital Gasthuisberg, University of Leuven, Leuven, Belgium; 7 Kon. Mathilde Moeder- en Kindcentrum, University Hospital of Antwerp, Antwerp, Belgium; 8 Academic Medical Center, Amsterdam, Netherlands; 9 Great Ormond Street Hospital, London, UK; 10 Maastricht University Medical Center, Maastricht, Netherlands; 11 Wilhelmina Children’s Hospital, University Medical Center Utrecht, Utrecht, Utrecht, Netherlands; 12 School of Medical Sciences, Faculty of Biology Medicine & Health, University of Manchester, Manchester, UK; 13 The Mark Holland Metabolic Unit, Salford Royal Foundation NHS Trust, Greater Manchester, M6 8HD, Salford, UK; 14 Willink Metabolic Unit, Manchester Centre for Genomic Medicine, Manchester University Hospitals NHS Foundation Trust, St Mary’s Hospital, Manchester, UK; 15 University of Leiden, Clinical Child and Adolescent Studies: Neurodevelopmental Disorders, Leiden, Netherlands

**Keywords:** Tyrosinemia type 1, Behavior problems, Health related-quality of life, Phenylalanine, Tyrosine

## Abstract

**Abstract:**

**Background:**

Treatment with 2-(2-nitro-4-trifluoromethylbenzoyl)-1,3-cyclohexanedione (NTBC) and dietary phenylalanine and tyrosine restriction improves physical health and life expectancy in Tyrosinemia type 1 (TT1). However, neurocognitive outcome is suboptimal. This study aimed to investigate behavior problems and health-related quality of life (HR-QoL) in NTBC-dietary-treated TT1 and to relate this to phenylalanine and tyrosine concentrations.

**Results:**

Thirty-one TT1 patients (19 males; mean age 13.9 ± 5.3 years) were included in this study. Emotional and behavioral problems, as measured by the Achenbach System of Empirically Based Assessment, were present in almost all domains. Attention and thought problems were particularly evident. HR-QoL was assessed by the TNO AZL Children’s and Adults QoL questionnaires. Poorer HR-QoL as compared to reference populations was observed for the domains: independent daily functioning, cognitive functioning and school performance, social contacts, motor functioning, and vitality. Both internalizing and externalizing behavior problems were associated with low phenylalanine (and associated lower tyrosine) concentrations during the first year of life. In contrast, high tyrosine (and associated higher phenylalanine) concentrations during life and specifically the last year before testing were associated with more internalizing behavior and/or HR-QoL problems.

**Conclusions:**

TT1 patients showed several behavior problems and a lower HR-QoL. Associations with metabolic control differed for different age periods. This suggests the need for continuous fine-tuning and monitoring of dietary treatment to keep phenylalanine and tyrosine concentrations within target ranges in NTBC-treated TT1 patients.

## Background

Tyrosinemia type 1 (TT1; McKusick 276,700) is an inborn error of tyrosine catabolism, caused by fumarylacetoacetate hydrolase deficiency. This causes accumulation of toxic metabolites which can result in liver failure and hepatocellular carcinoma (HCC), renal tubular dysfunction and neurological porphyria-like crises [1]. Before the 1990s, dietary restriction of tyrosine and its precursor phenylalanine was the only treatment to reduce the synthesis of toxic metabolites, while not preventing liver complications. Consequently, life expectancy was very poor and liver transplantation was the only definitive treatment option [2].

In 1992, 2-(2-nitro-4-trifluoromethylbenzoyl)-1,3-cyclohexanedione (NTBC) was introduced as a new treatment [3]. NTBC blocks the tyrosine degradation pathway upstream from the primary defect, thereby preventing the formation of toxic metabolites, but also leading to higher tyrosine concentrations. Therefore, dietary restriction of phenylalanine and tyrosine remains necessary. The use of NTBC has undoubtedly improved outcome and life expectancy, preventing both hepatic and extrahepatic problems [4].

However, recent studies suggest that the neurocognitive outcome of NTBC-dietary-treated TT1 patients is suboptimal [5–11]. Lower IQ values have been observed along with impairment in various cognitive domains, including executive functioning and social cognition. These neurocognitive defects may underlie problems in TT1 patients’ daily lives, such as school problems and attention deficits. At present, knowledge of the cognitive-behavioral phenotype associated with TT1 and its cause is limited, although high tyrosine and low phenylalanine concentrations may both be associated with brain dysfunction [5, 8, 12–14].

The present study aimed to investigate the emotional and behavioral phenotype of TT1 patients and to look for associations with biochemical parameters in a relatively large patient group. First, we assessed the emotional and behavioral problems and health-related quality of life (HR-QoL) of TT1 patients. Second, we investigated the relationship between these outcomes and plasma phenylalanine and tyrosine concentrations.

## Methods

### Participants

In total, 31 TT1 patients (19 males) were included in this cross-sectional study (mean age 13.9 ± 5.3 years) (Table 1). Patients <six years and/or patients who received liver transplantation were excluded. All patients were treated with NTBC (typically at a dose of 1 mg/kg body weight/day) and a phenylalanine-tyrosine-restricted diet. Eleven patients had (or still) received phenylalanine supplementation at the time of assessment because phenylalanine concentrations were below the recommended limit of 30 μmol/L [12]. Patients were included from different centers in the UK, Belgium, and the Netherlands between October 2012 and August 2018. Plasma phenylalanine and tyrosine concentrations were collected from patient records. The study was approved by the Medical Ethical Committees of the participating centers. All patients and/or parents gave informed consent to participate in this study.

**Table 1:** Patient characteristics

Patient	Age (years)	Time of diagnosis (pre-symptomatic, < 2 months, 2–6 months, > 6 months)	Phenylalanine supplementation	Median first year concentrations		Median lifetime concentrations		Median last year concentrations	
				Phe (μmol/L)	Tyr (μmol/L)	Phe (μmol/L)	Tyr (μmol/L)	Phe (μmol/L)	Tyr (μmol/L)
1	6.6	< 2 months	No	10	195.5	43.5	512.5	44	518
2	7.3	> 6 months	Yes	–	–	–	–	29	344
3	7.9	> 6 months	No	–	–	42	389	41	521.5
4	8.1	Pre-symptomatic	No	29.5	342.5	32.5	295	37	405
5	8.1	> 6 months	No	–	–	41	483.9	44	462
6	8.3	2–6 months	Yes	28	257	19	376	18	401
7	8.8	Pre-symptomatic	Yes	18	245	20	490	32	759.5
8	9.2	Pre-symptomatic	Yes	26	281	21	457	39	514
9	9.7	2–6 months	No	40	245	26	397	38	427
10	9.9	2–6 months	Yes	16.5	155	27.8	370	45	572
11	10.3	Pre-symptomatic	Yes	18	180	22	330	52	506
12	10.4	Pre-symptomatic	Yes	18	280	31	370	60.5	615.5
13	10.6	Pre-symptomatic	Yes	–	–	21	259	26	349
14	12.0	Pre-symptomatic	No	26	448	–	518	30	477
15	12.1	Pre-symptomatic	Yes	24	286	22	286	46.5	374
16	13.4	2–6 months	No	43	300	30	407	–	–
17	14.0	Pre-symptomatic	No	55	538	47	668.3	52	959
18	15.1	< 2 months	Yes	61.5	372	24.5	542.5	67	746
19	15.4	Pre-symptomatic	No	25	365.5	18	530.5	60	766
20	15.6	Pre-symptomatic	No	39	643	42.5	628.5	45.5	700
21	16.0	2–6 months	No	22.5	293	36.5	398.5	17	382
22	16.5	< 2 months	No	24	366.5	28	392	38	434
23	16.9	Pre-symptomatic	No	41	361.5	46.3	713.5	64	866
24	17.7	2–6 months	No	67	631	48	573	58	735.5
25	18.4	Pre-symptomatic	No	22	351	10	509.5	–	–
26	19.5	Pre-symptomatic	No	41.5	663.5	20	625.3	–	–
27	20.5	< 2 months	No	–	–	–	–	45.5	620.5
28^a^	22.5	Pre-symptomatic	No	–	–	–	–	44	248
29^b^	23.0	2–6 months	No	43	412.5	44.3	689	55	766
30^c^	23.3	> 6 months	Yes	60	43	42	306.8	45	280
31^a^	23.5	> 6 months	No	10	195.5	44.5	483	58	484

Patients that did not receive NTBC treatment directly after diagnosis are indicated with asterisks. ^a^NTBC started approximately 1 month after diagnosis ^b^NTBC started < 1 year after diagnosis ^c^NTBC started > 1 year after diagnosis

### Instruments

To assess emotional and behavioral problems, the Achenbach System of Empirically Based Assessment (ASEBA) questionnaires were used [15, 16]. The Child Behavior Checklist (CBCL), suitable for age 6–12 years, was completed by parents. Adolescents (13–17 years) and adults (≥18 years) filled out the Youth Self Report (YSR) or the Adult Self Report (ASR). The CBCL, YSR, and ASR measure emotional and behavioral problems in seven different dimensions: withdrawn/depressed, somatic complaints, anxious/depressed, thought problems, attention problems, rule-breaking behavior, and aggressive behavior. Additionally, the CBCL and YSR assess social problems, whereas the ASR assesses intrusive behavior. The sum of scores on dimensions withdrawn/depressed, somatic complaints, and anxious/depressed forms the broadband scale ‘internalizing problems’, while the broadband scale ‘externalizing problems’ is based on the rule-breaking, aggressive, and (in case of the ASR) intrusive behavior dimensions [15, 16]. Furthermore, six Diagnostic and Statistical Manual of Mental Disorders (DSM) IV oriented scales were used. Different scales were used for the CBCL and YSR (affective, anxiety, somatic, attention deficit hyperactivity, oppositional, and conduct problems), and the ASR (depressive, anxiety, somatic, avoidant personality, attention deficit hyperactivity, and antisocial personality problems). For all ASEBA questionnaires, higher scores indicate more problems in that domain.

To assess HR-QoL, the TNO AZL Children’s Quality Of Life (TACQOL) questionnaire was used for children aged < 16 years [17]. For analysis, this questionnaire was divided into two parts: 1) for children aged 8–11 and 2) for children aged 12–15 years. Both questionnaires included 63 items and were filled out by the parents. For children aged 8–11, the TACQOL questionnaire consisted of seven different dimensions measuring physical functioning/complaints (‘body’), motor functioning (‘motor’), independent daily functioning (‘autonomy’), cognitive functioning and school performances (‘cognition’), social contacts with parents and peers (‘social’), occurrence of positive moods (‘positive emotions’), and of negative moods (‘negative emotions’). For children aged 12–15, the dimensions autonomy and social were combined, measuring interaction with peers (‘peers’). For participants aged ≥16 years, the TNO AZL Adult’s Quality Of Life (TAAQOL) questionnaire was used; this consisted of 45 items. The TAAQOL included 12 different dimensions, i.e. gross motor functioning (‘gross motor’), fine motor functioning (‘fine motor’), cognitive functioning (‘cognition’), sleep quality (‘sleep’), pain (‘pain’), social functioning (‘social’), independent daily functioning (‘daily activities’), sexuality (‘sex’), vitality (‘vitality’), positive moods (‘happiness’), depressive moods (‘depressive’), and angry moods (‘anger’). For all questionnaires, a higher score indicated a better HR-QoL.

The HR-QoL data of TT1 patients was compared to a Dutch reference population divided into 3 reference groups of participants aged 8–11 years (*N* = 548), aged 12–15 (*N* = 393), and aged 16–30 years (*N* = 394).

### Biochemical data

Patient data were collected from clinical records to investigate differences within the TT1 group. Additionally, all phenylalanine and tyrosine concentrations were retrieved from the clinical patient records. Dried blood spot samples, when stated as such in the patient files, were excluded in order to only include venous samples in our analysis. In general, three to four venous samples were obtained every year. Based on the existing literature on associations between metabolic control and behavioral outcomes in TT1 and phenylketonuria we focused on phenylalanine and tyrosine levels during three periods in life: the first year after birth, throughout lifetime (until assessment), and the last year before assessment [5, 8, 13, 18, 19]. Since some plasma phenylalanine concentrations < 30 μmol/L were unspecified, imputation was used to allow inclusion of these concentrations in analyses. For the imputation, median phenylalanine concentrations < 30 μmol/L were calculated using available data from other patients in whom the plasma concentrations < 30 μmol/L were specified. Lifetime concentrations were calculated as the median of yearly median phenylalanine and tyrosine levels.

### Statistical analyses

For the analysis of emotional and behavioral problems, raw ASEBA-scores were converted into T-scores to allow comparison to the general population and between different groups of TT1 patients. Descriptive analyses were performed using pre-defined T-scores indicating scores in the normal range (50–64), borderline range (65–69) and clinical range (≥70) for all different subdomains of the ASEBA questionnaires, and borderline range (60–63), and clinical range (64–100) for internalizing problems and externalizing problems [15, 16]. The borderline range indicates that scores are not clearly deviant from norm scores, but high enough to be of concern. Scores in the clinical range indicate a deviation from norm scores, and therefore possible clinical importance which may warrant intervention. For the assessment of HR-QoL, TT1 patients were compared with Dutch reference data using Mann-Whitney U tests. Kruskal Wallis tests and/or Mann-Whitney U tests were performed to analyze differences within the TT1 group, comparing pre-symptomatically versus symptomatically diagnosed patients, and symptomatically diagnosed patients diagnosed at different ages (< 2 months, 2–6 months or > 6 months of age [2]). Spearman correlation tests were performed to study associations between behavior problems and HR-QoL, and between behavior problems and HR-QoL-outcomes on the one hand and plasma phenylalanine and tyrosine concentrations on the other. The broadband ASEBA-scales were excluded from these last correlational analyses since these were calculated using the subdomains and therefore significant results would be expected when subdomains were statistically significant. All statistical analyses were performed using IBM SPSS Statistics 22nd version and *p* < 0.05 was considered statistically significant.

## Results

### Emotional and behavioral problems

Table 2 summarizes the findings of the ASEBA questionnaires. When considering internalizing problems, 47% of children, 22% of adolescents, and 14% of adults with TT1 scored within the borderline or clinical range. When considering externalizing problems, 54% of children, 22% of adolescents and no adults scored within the borderline or clinical range. TT1 children showed problems in all domains, but especially with respect to attention problems, with 53% being in clinical range. Adolescents and adults especially showed thought and attention problems, respectively. When investigating differences between the different age groups using Kruskal-Wallis analysis, children showed significantly more attention problems than adults (*p* = 0.025). There were no significant differences in behavior problems observed between symptomatically and pre-symptomatically diagnosed patients.

**Table 2:** Results of the empirical and the DSM-IV oriented scales of the ASEBA questionnaires

	Children (8–12) (*N* = 15, 9 males)		Adolescents (13–17) (N = 9, 6 males)		Adults (≥18) (*N* = 7, 4 males)	
	Borderline range (%)	Clinical range (%)	Borderline range (%)	Clinical range (%)	Borderline range (%)	Clinical range (%)
ASEBA empirical scales						
Withdrawn/ depressed	20	7	11	0	14	0
Somatic complaints	7	27	0	11	0	0
Anxious/ depressed	27	7	0	11	0	0
Social problems	7	27	11	11	–	–
Thought problems	13	20	11	11	0	29
Attention problems	0	53	44	0	0	0
Rule-breaking behavior	13	27	0	11	0	14
Aggressive behavior	27	13	0	11	0	14
Intrusive behavior	–	–	–	–	0	0
Internalizing problems	7	40	11	11	14	0
Externalizing problems	7	47	11	11	0	0
ASEBA DSM-IV oriented scales						
Affective problems	7	33	11	11	–	–
Depressive problems	–	–	–	–	0	0
Anxiety problems	20	7	11	11	0	0
Somatic problems	13	27	0	11	29	0
Avoidant personality problems	–	–	–	–	0	14
Attention deficit hyperactivity problems	13	40	22	0	14	0
Oppositional problems	13	7	0	22	–	–
Conduct problems	27	20	0	11	–	–
Antisocial personality problems	–	–	–	–	0	0

Internalizing problems are based on the withdrawn/depressed, somatic complaints, and anxious/depressed domains, and externalizing problems are based on rule-breaking, aggressive, and (in case of the ASR) intrusive behavior domains

Table 2 also shows the descriptive findings on the DSM-IV oriented scales of the ASEBA. TT1 patients reported problems on most scales, especially affective, somatic, attention deficit hyperactivity, and conduct issues. Although not statistically significant, scores within the borderline or clinical range were generally observed more frequently in children than in adolescents and adults.

### Health-related quality of life

**Fig. 1:**
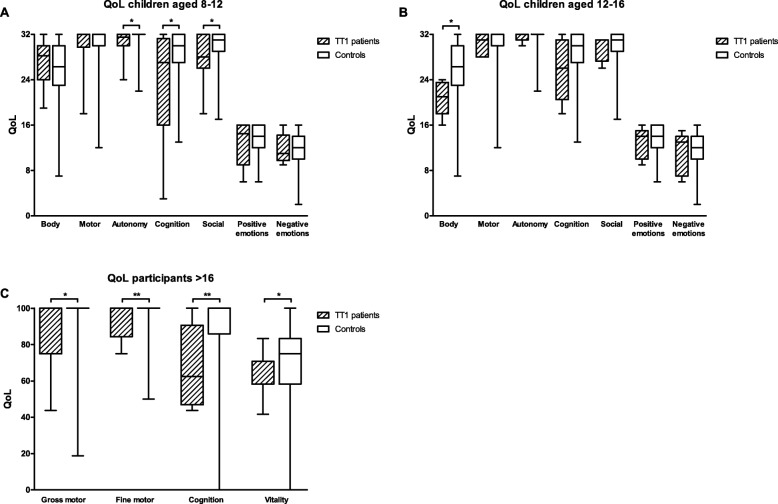
HR-QoL of TT1 patients compared to controls. **a**. TACQOL for 8-11 year old children compared to controls (*N*=14 patients). **b**. TACQOL for 12-15 year old children compared to controls (*N*=5 patients). **c**. TAAQOL for patients ≥16 years old compared to controls (*N*=9 patients). Boxes indicate 25-75 percentile, whiskers indicate min-max. * *p*<0.05, ** *p*<0.01

Figure 1a shows the results of the HR-QoL questionnaires of TT1 children compared to the Dutch reference population. The TACQOL questionnaire for children aged 8–11 showed a significantly lower HR-QoL for the domains autonomy (*p* = 0.011), cognition (*p* = 0.029) and social (*p* = 0.039) in TT1 patients. When comparing the TACQOL scores for TT1 children aged 12–15 with the corresponding reference population, a significantly lower HR-QoL was observed on the domain body (*p* = 0.014) (Fig. 1b). For both age groups (8–11 and 12–15), analyses of the TACQOL showed no significant differences between pre-symptomatically and symptomatically diagnosed patients, and no differences between the different times of diagnosis.

Figure 1c shows that adult TT1 patients (*N* = 9) reported a significantly lower HR-QoL in the domains gross motor (0.016), fine motor (*p* < 0.001), cognition (*p* = 0.001), and vitality (*p* = 0.046). When comparing pre-symptomatically (*N* = 3) and symptomatically (*N* = 6) diagnosed patients, a significant difference was observed on the domain vitality (*p* = 0.024), indicating a lower HR-QoL in pre-symptomatically diagnosed patients. When comparing groups based on age at diagnosis, no significant differences were observed.

HR-QoL and emotional/behavioral problems were often significantly correlated (see Additional files 1 and 2).

### Phenylalanine and tyrosine associated to each other

As phenylalanine is a metabolic precursor of tyrosine, a relationship between phenylalanine and tyrosine concentrations is expected. Phenylalanine and tyrosine concentrations during the first year of life were, indeed, positively correlated (ρ = 0.532; *p* = 0.007), as were the concentrations during the last year (ρ = 0.631; *p* < 0.001). Lifetime concentrations showed a positive trend (*ρ* = 0.329; *p* = 0.094).

### Associations of outcomes with first year plasma phenylalanine and tyrosine concentrations

Significant negative correlations were observed between first year phenylalanine concentrations and somatic complaints (*ρ* = − 0.421; *p* = 0.040), social problems (*ρ* = − 0.457; *p* = 0.043), thought problems (*ρ* = − 0.430; *p* = 0.040), and attention problems (*ρ* = − 0.513; *p* = 0.010). Furthermore, negative correlations were observed between first year tyrosine concentrations and social problems (*ρ* = − 0.608; *p* = 0.004), attention problems (*ρ* = − 0.598; *p* = 0.002), delinquent behavior (*ρ* = − 0.417; *p* = 0.043), and aggressive behavior (*ρ* = − 0.438; *p* = 0.032). This indicates that low phenylalanine (and the associated lower tyrosine) concentrations during the first year of life were associated with both internalizing and externalizing behavior problems.

The DSM-IV oriented scales showed negative correlations between first year tyrosine concentrations and affective problems (*ρ* = − 0.449; *p* = 0.047), attention deficit hyperactivity problems (*ρ* = − 0.493; *p* = 0.014), and conduct problems (*ρ* = − 0.667; *p* = 0.001).

Results for the HR-QoL in children, showed a positive correlation between first year plasma phenylalanine concentrations and autonomy (*ρ* = 0.609; *p* = 0.016). In adults, a negative correlation was observed between first year phenylalanine concentrations and cognition (*ρ* = − 0.943; *p* = 0.005). Results of the correlation analyses are also summarized in Additional file 3.

### Associations of outcomes with lifetime plasma phenylalanine and tyrosine concentrations

No significant correlations were observed between results on the ASEBA questionnaires and lifetime plasma phenylalanine and tyrosine concentrations. Correlational analyses with the HR-QoL questionnaires showed a positive correlation between lifetime phenylalanine concentrations and autonomy (ρ = 0.517; *p* = 0.034) in children, indicating more autonomy with higher (normal rather than low) phenylalanine concentrations. Furthermore, negative correlations were observed between lifetime phenylalanine concentrations and cognition (ρ = − 0.829; *p* = 0.021) and social contacts (ρ = − 0.802; *p* = 0.030) in patients aged > 16 years, indicating better HR-QoL with lower phenylalanine concentrations (also see Additional file 3).

### Associations of outcomes with last year plasma phenylalanine and tyrosine concentrations

A significant correlation was observed between the last year phenylalanine concentrations and the score in the withdrawn/depressed domain (*ρ* = 0.411; *p* = 0.030) indicating more problems with higher phenylalanine concentrations. The last year tyrosine concentrations also correlated with the withdrawn/depressed score (*ρ* = 0.492; *p* = 0.008), indicating more problems with higher tyrosine concentrations. When investigating the DSM-oriented ASEBA scales, higher phenylalanine concentrations correlated with increased affective problems (*ρ* = 0.418; *p* = 0.047) and anxiety problems (*ρ* = 0.381; *p* = 0.045). These findings indicate that higher (usually normal) phenylalanine concentrations and the associated higher tyrosine concentrations are associated with more problems.

Results for the HR-QoL in children, showed a significant negative correlation between the last year tyrosine concentrations and positive emotions (*ρ* = − 0.505; *p* = 0.033), indicating more positive emotions with lower (towards normal rather than high) tyrosine levels. In adult patients, the last year phenylalanine concentrations negatively correlated with the score in the social domain (*ρ* = − 0.802; *p* = 0.030) (also see Additional file 3).

## Discussion

In this study, we investigated emotional and behavioral problems and HR-QoL in TT1 patients, and their relation to plasma phenylalanine and tyrosine concentrations. The most important findings were that TT1 patients showed behavior problems and lower HR-QoL on several domains compared to the Dutch reference population used. Furthermore, several correlations were observed between these neurocognitive impairments and metabolic control. Most interestingly, correlations with low plasma phenylalanine concentrations during the first year(s) of life and high plasma tyrosine concentrations later in life were observed.

With respect to behavior problems, in addition to the attention problems that were identified previously [11], our results showed TT1 patients treated with NTBC and diet experienced a number of other behavior problems including rule-breaking behavior, social problems, and somatic complaints. On these domains, > 25% of the children scored within the clinical range. Overall, both internalizing and externalizing behavior problems were found. As identified with the HR-QoL assessments, TT1 children described problems in cognitive functioning and school performance, social contacts with parents and peers and independent daily functioning. TT1 adults had problems with gross and fine motor functioning, cognition, and vitality.

Our results substantiate previous studies on neurocognitive problems using other methods [5–8, 10, 11]. Although chronic illness may directly affect HR-QoL [20], results on the ASEBA and HR-QoL questionnaires were often associated, suggesting that chronic illness could also affect HR-QoL indirectly, through behavior problems. Thus, our results stressed the importance of addressing both behavioral problems and HR-QoL problems in daily patient care.

Furthermore, we investigated correlations with phenylalanine and tyrosine concentrations as a possible cause of the observed problems, which has not been studied in such detail before. High phenylalanine and tyrosine levels may have toxic effects on the brain, as shown in disorders such as phenylketonuria and tyrosinemia type 2 [18, 21–23]. There is also evidence that low plasma phenylalanine concentrations adversely affect the outcomes in PKU [24] and TT1 [5, 12, 13]. It is important to note that low plasma phenylalanine concentrations in TT1 are likely to cause even lower phenylalanine concentrations in the brain because of competition with tyrosine for influx across the blood-brain barrier. This competitive inhibition has also been observed in PKU where high phenylalanine concentrations inhibit the influx of tyrosine [25]. Low cerebral concentrations of any limiting essential amino acid may impair cerebral protein synthesis [26].

The results of this study indicate that, during the first year of life, behavior problems correlate with low phenylalanine rather than high tyrosine concentrations. This changes later in life when, during last year before testing, high tyrosine concentrations correlate with more behavior problems (although they are associated with higher phenylalanine levels). An attractive explanation for this time dependency would be that while low phenylalanine concentrations during the first year are detrimental as a phenylalanine shortage impedes the rapidly developing brain, at a later age the high tyrosine concentrations may be more likely to have long term chronic toxic effects. Further research is, however, necessary to investigate this hypothesis. Moreover, the relationship between amino acid levels and behavioral outcomes may be non-linear, or only linear within certain ranges: both too low and too high amino acid levels may be detrimental.

This study has a number of limitations, many of them due to the rarity of TT1, affecting approximately 1:100.000 newborns. Although inclusion of 31 patients may be considered quite good, it is impossible to reach high statistical power with such numbers, especially since our patient sample was rather heterogeneous, varying in age, age at diagnosis, and symptoms at presentation. Moreover, patients were recruited from three different countries, but for HR-QoL only Dutch reference data were available. We have, therefore, been cautious of analyzing the influence of the aforementioned variables on behavior/HR-QoL-outcomes. Furthermore, the direct influence of NTBC could not be investigated. Moreover, blood samples were taken under different conditions (fasting/non-fasting) and at different time points. Next to this, the effect of phenylalanine supplementation and the different dosages, which could have affected the phenylalanine and tyrosine concentrations, could not be investigated in this study. Centers may differ in plasma analysis methods and number of samples taken per patients.

## Conclusion

To conclude, TT1 patients have more emotional and behavioral problems, and a lower HR-QoL than healthy individuals. The impairments in behavior and HR-QoL were partly related to metabolic control, with low phenylalanine and tyrosine levels in the first year of life and high recent phenylalanine and tyrosine levels being related to poorer outcomes. Whereas neonatal screening and early treatment with NTBC have dramatically improved physical health and life expectancy in TT1, the data presented in this study underline the need to optimize dietary treatment to improve emotional and behavioral outcomes in this population.

## Supplementary information

**Additional file 1. **Correlations between ASEBA questionnaires and QoL questionnaires for children. Correlations between ASEBA questionnaires and QoL questionnaires for children (*N* = 19). Bold and underlined results are significant correlations. The QoL domain peers only consists of children aged 12–15 years (*N* = 5).

**Additional file 2. **Correlations between ASEBA questionnaires and QoL questionnaires for patients aged 16 and older. Correlations between ASEBA questionnaires and QoL questionnaires for patients aged 16 and older (*N* = 9). Bold and underlined results are significant correlations. For patients 16–18 years old (*N* = 2), the ASEBA questionnaire did not calculated the scales intrusive, depressed, avoidant and antisocial. The domain sex could not be calculated for one of the patients due to missing answers in the questionnaire.

**Additional file 3. **Summarized results of correlation analyses between phenylalanine and tyrosine concentrations and neurocognitive outcome scores. Summarized results of correlation analyses between phenylalanine and tyrosine concentrations and neurocognitive outcome scores. Only scales with significant correlations, with *p*-values < 0.05, are shown. *ρ* = Spearman’s rho. For ASEBA, positive correlations indicate that higher levels are related to poorer outcomes, whereas negative correlations indicate that higher levels are related to better outcomes; for HR-QoL, positive correlations indicate that higher levels are related to better outcomes, whereas negative correlations indicate that higher levels are related to poorer outcomes.

## Data Availability

The datasets used and/or analysed during the current study are available from the corresponding author on reasonable request.

## Abbreviations

ASEBA: Achenbach System of Empirically Based Assessment
ASR: Adult Self Report
CBCL: Child Behavior Checklist
DSM-IV: Diagnostic and Statistical Manual of Mental Disorders IV
HCC: Hepatocellular carcinoma
HR-QoL: Health-Related Quality of Life
NTBC: 2-(2-nitro-4-trifluoromethylbenoyl)-1,3-cyclohexanedione
TAAQOL: TNO AZL Adult’s Quality Of Life
TACQOL: TNO AZL Children’s Quality Of Life
TT1: Tyrosinemia type 1
YSR: Youth Self Report
