# Supplementary material for: Emotional and behavioral problems, quality of life and metabolic control in NTBC-treated Tyrosinemia type 1 patients
Source: Orphanet J Rare Dis. 2019 Dec 4;14:285. doi: 10.1186/s13023-019-1259-2 (PMC6894144; doi:10.1186/s13023-019-1259-2)
Supplement: Supplementary file 1 — Additional file 1. Correlations between ASEBA questionnaires and QoL questionnaires for children. Correlations between ASEBA questionnaires and QoL questionnaires for children (N = 19). Bold and underlined results are significant correlations. The QoL domain peers only consists of children aged 12–15 years (N = 5). [file 13023_2019_1259_MOESM1_ESM.docx]

**Additional file 1.** Correlations between ASEBA questionnaires and QoL questionnaires for children.

|  | Body | Motor | Autonomy | Cognition | Social | Positive emotions | Negative emotions | Peers |
| --- | --- | --- | --- | --- | --- | --- | --- | --- |
| ASEBA scales |  |  |  |  |  |  |  |  |
| Withdrawn/depressed  Rho (ρ) =  *p* =  N = | 0.021  0.933  19 | -0.252  0.297  19 | -0.370  0.119  19 | -0.379  0.109  19 | -0.224  0.356  19 | **-0.741**  **0.000**  **19** | -0.284  0.238  19 | -0.447  0.450  5 |
| Somatic  ρ =  *p* =  N = | -0.113  0.645  19 | -0.289  0.230  19 | 0.131  0.594  19 | 0.015  0.953  19 | -0.071  0.772  19 | -0.359  0.131  19 | -0.320  0.181  19 | 0.112  0.858  5 |
| Anxious/depressed  ρ =  *p* =  N = | -0.058  0.814  19 | -0.240  0.322  19 | **-0.516**  **0.024**  **19** | -0.267  0.269  19 | -0.267  0.269  19 | **-0.632**  **0.004**  **19** | **-0.576**  **0.010**  **19** | -0.125  0.841  5 |
| Social  ρ =  *p* =  N = | 0.101  0.680  19 | 0.124  0.612  19 | **-0.495**  **0.031**  **19** | -0.421  0.073  19 | -0.418  0.075  19 | **-0.586**  **0.008**  **19** | -0.427  0.068  19 | 0.115  0.854  5 |
| Thought  ρ =  *p* =  N = | -0.116  0.647  18 | -0.161  0.522  18 | **-0.532**  **0.023**  **18** | -0.397  0.103  18 | -0.399  0.101  18 | **-0.486**  **0.041**  **18** | **-0.558**  **0.016**  **18** | 0.112  0.858  5 |
| Attention  ρ =  *p* =  N = | 0.036  0.883  19 | 0.098  0.691  19 | -0.334  0.163  19 | **-0.697**  **0.001**  **19** | **-0.569**  **0.011**  **19** | -0.360  0.130  19 | -0.291  0.226  19 | -0.344  0.571  5 |
| Rule-breaking  ρ =  *p* =  N = | 0.109  0.657  19 | 0.075  0.762  19 | -0.128  0.600  19 | -0.110  0.654  19 | -0.087  0.722  19 | -0.392  0.096  19 | -0.150  0.541  19 | 0.344  0.571  5 |
| Aggressive  ρ =  *p* =  N = | 0.205  0.401  19 | 0.013  0.959  19 | -0.107  0.664  19 | -0.194  0.425  19 | -0.366  0.123  19 | -0.402  0.088  19 | -0.392  0.097  19 | 0.344  0.571  5 |
| Internalizing  ρ =  *p* =  N = | -0.151  0.538  19 | -0.421  0.073  19 | -0.331  0.166  19 | -0.355  0.136  19 | -0.279  0.248  19 | **-0.702**  **0.001**  **19** | **-0.575**  **0.010**  **19** | -0.344  0.571  5 |
| Externalizing  ρ =  *p* =  N = | 0.237  0.329  19 | 0.065  0.791  19 | -0.150  0.541  19 | -0.160  0.513  19 | -0.279  0.247  19 | **-0.503**  **0.028**  **19** | -0.301  0.210  19 | 0.224  0.718  5 |
|  |  |  |  |  |  |  |  |  |
|  | **Body** | **Motor** | **Autonomy** | **Cognition** | **Social** | **Positive emotions** | **Negative emotions** | **Peers** |
| ASEBA DSM scales |  |  |  |  |  |  |  |  |
| Affective  ρ =  *p* =  N = | -0.009  0.971  19 | -0.184  0.451  19 | -0.316  0.187  19 | -0.235  0.333  19 | -0.314  0.190  19 | **-0.564**  **0.012**  **19** | **-0.605**  **0.006**  **19** | 0.000  1.000  5 |
| Anxiety  ρ =  *p* =  N = | -0.360  0.130  19 | -0.205  0.399  19 | -0.438  0.061  19 | -0.302  0.209  19 | -0.264  0.276  19 | -0.432  0.065  19 | **-0.518**  **0.023**  **19** | 0.000  1.000  5 |
| Somatic  ρ =  *p* =  N = | -0.162  0.508  19 | -0.371  0.118  19 | 0.379  0.110  19 | 0.101  0.681  19 | 0.021  0.933  19 | -0.040  0.869  19 | -0.208  0.393  19 | -0.229  0.710  5 |
| Attention deficit hyperactivity  ρ =  *p* =  N = | -0.063  0.797  19 | 0.175  0.474  19 | -0.072  0.769  19 | **-0.513**  **0.025**  **19** | **-0.462**  **0.046**  **19** | -0.119  0.627  19 | -0.238  0.327  19 | 0.000  1.000  5 |
| Oppositional  ρ =  *p* =  N = | -0.010  0.968  19 | -0.044  0.859  19 | 0.021  0.933  19 | -0.148  0.546  19 | -0.378  0.110  19 | -0.245  0.312  19 | -0.398  0.091  19 | 0.344  0.571  5 |
| Conduct  ρ =  *p* =  N = | 0.302  0.209  19 | 0.100  0.683  19 | -0.078  0.750  19 | -0.122  0.619  19 | -0.144  0.557  19 | **-0.564**  **0.012**  **19** | -0.147  0.548  19 | 0.344  0.571  5 |

Correlations between ASEBA questionnaires and QoL questionnaires for children (N=19). Bold and underlined results are significant correlations. The QoL domain peers only consists of children aged 12-15 years (N=5).
