# Supplementary material for: Emotional and behavioral problems, quality of life and metabolic control in NTBC-treated Tyrosinemia type 1 patients
Source: Orphanet J Rare Dis. 2019 Dec 4;14:285. doi: 10.1186/s13023-019-1259-2 (PMC6894144; doi:10.1186/s13023-019-1259-2)
Supplement: Supplementary file 2 — Additional file 2. Correlations between ASEBA questionnaires and QoL questionnaires for patients aged 16 and older. Correlations between ASEBA questionnaires and QoL questionnaires for patients aged 16 and older (N = 9). Bold and underlined results are significant correlations. For patients 16–18 years old (N = 2), the ASEBA questionnaire did not calculated the scales intrusive, depressed, avoidant and antisocial. The domain sex could not be calculated for one of the patients due to missing answers in the questionnaire. [file 13023_2019_1259_MOESM2_ESM.docx]

**Additional file 2.** Correlations between ASEBA questionnaires and QoL questionnaires for patients aged 16 and older.

|  | Gross motor | Fine motor | Cognition | Sleep | Pain | Social contacts | Daily activities | Sex | Vitality | Positive | Depressive | Aggressive |
| --- | --- | --- | --- | --- | --- | --- | --- | --- | --- | --- | --- | --- |
| ASEBA empirical scales |  |  |  |  |  |  |  |  |  |  |  |  |
| Withdrawn/depressed  Rho (ρ) =  *p* =  N = | -0.088  0.822  9 | 0.079  0.840  9 | -0.501  0.170  9 | -0.214  0.581  9 | 0.148  0.704  9 | **-0.753**  **0.019**  **9** | -0.176  0.651  9 | -0.366  0.373  8 | -0.337  0.376  9 | -0.665  0.051  9 | **-0.915**  **0.001**  **9** | -0.636  0.066  9 |
| Somatic  ρ =  *p* =  N = | -0.321  0.400  9 | -0.541  0.132  9 | 0.374  0.321  9 | 0.240  0.533  9 | **-0.754**  **0.019**  **9** | 0.289  0.451  9 | -0.321  0.400  9 | -0.290  0.486  8 | -0.179  0.644  9 | 0.068  0.863  9 | 0.147  0.707  9 | 0.138  0.724  9 |
| Anxious/depressed  ρ =  *p* =  N = | -0.152  0.697  9 | -0.136  0.727  9 | -0.449  0.226  9 | -0.060  0.878  9 | 0.125  0.749  9 | **-0.779**  **0.013**  **9** | 0.182  0.640  9 | -0.219  0.602  8 | -0.167  0.667  9 | -0.440  0.235  9 | **-0.867**  **0.002**  **9** | -0.426  0.254  9 |
| Thought  ρ =  *p* =  N = | -0.141  0.718  9 | -0.010  0.979  9 | -0.245  0.526  9 | -0.093  0.811  9 | -0.320  0.401  9 | -0.303  0.428  9 | 0.169  0.664  9 | -0.166  0.695  8 | -0.263  0.494  9 | -0.536  0.137  9 | **-0.839**  **0.005**  **9** | **-0.682**  **0.043**  **9** |
| Attention  ρ =  *p* =  N = | -0.158  0.685  9 | -0.253  0.511  9 | -0.202  0.602  9 | 0.305  0.425  9 | -0.423  0.527  9 | -0.083  0.831  9 | -0.316  0.407  9 | 0.587  0.126  8 | 0.361  0.339  9 | 0.070  0.859  9 | -0.418  0.263  9 | -0.578  0.103  9 |
| Delinquent  ρ =  *p* =  N = | -0.226  0.559  9 | -0.188  0.628  9 | -0.517  0.154  9 | -0.028  0.943  9 | -0.156  0.688  9 | -0.550  0.125  9 | 0.071  0.857  9 | 0.111  0.793  8 | 0.100  0.799  9 | -0.248  0.520  9 | **-0.825**  **0.006**  **9** | -0.613  0.079  9 |
| Aggressive  ρ =  *p* =  N = | -0.224  0.562  9 | -0.101  0.797  9 | -0.162  0.676  9 | -0.111  0.775  9 | -0.283  0.460  9 | -0.395  0.293  9 | 0.056  0.886  9 | -0.302  0.467  8 | -0.412  0.270  9 | **-0.729**  **0.026**  **9** | **-0.926**  **0.000**  **9** | -0.652  0.057  9 |
| Intrusive  ρ =  *p* =  N = | 0.196  0.674  7 | 0.025  0.958  7 | 0.361  0.426  7 | 0.184  0.693  7 | -0.225  0.628  7 | 0.516  0.236  7 | 0.685  0.090  7 | 0.287  0.581  6 | -0.071  0.880  7 | 0.512  0.240  7 | 0.408  0.364  7 | 0.294  0.523  7 |
| Internalizing  ρ =  *p* =  N = | -0.196  0.613  9 | -0.211  0.585  9 | -0.282  0.462  9 | 0.056  0.887  9 | -0.204  0.599  9 | -0.604  0.085  9 | -0.140  0.719  9 | -0.280  0.503  8 | -0.283  0.460  9 | -0.525  0.146  9 | **-0.866**  **0.003**  **9** | -0.572  0.108  9 |
| Externalizing  ρ =  *p* =  N = | -0.358  0.344  9 | -0.337  0.376  9 | -0.303  0.429  9 | 0.018  0.963  9 | -0.331  0.385  9 | -0.479  0.192  9 | -0.055  0.888  9 | 0.000  1.000  8 | 0.008  0.983  9 | -0.433  0.244  9 | **-0.860**  **0.003**  **9** | -0.597  0.089  9 |
|  |  |  |  |  |  |  |  |  |  |  |  |  |
|  | **Gross motor** | **Fine motor** | **Cognition** | **Sleep** | **Pain** | **Social contacts** | **Daily activities** | **Sex** | **Vitality** | **Positive** | **Depressive** | **Aggressive** |
| ASEBA DSM-IV oriented scales |  |  |  |  |  |  |  |  |  |  |  |  |
| Depressed  ρ =  *p* =  N = | -0.175  0.707  7 | -0.420  0.348  7 | -0.410  0.361  7 | 0.019  0.967  7 | 0.485  0.269  7 | **-0.840**  **0.018**  **7** | 0.434  0.331  7 | -0.250  0.633  6 | 0.144  0.758  7 | 0.019  0.968  7 | -0.576  0.176  7 | 0.154  0.742  7 |
| Anxious  ρ =  *p* =  N = | -0.151  0.699  9 | -0.130  0.739  9 | -0.267  0.487  9 | 0.020  0.959  9 | -0.105  0.788  9 | -0.525  0.147  9 | 0.181  0.641  9 | -0.058  0.891  8 | -0.203  0.600  9 | -0.493  0.178  9 | **-0.877**  **0.002**  **9** | -0.558  0.118  9 |
| Somatic  ρ =  *p* =  N = | 0.000  1.000  9 | -0.171  0.660  9 | 0.615  0.078  9 | 0.390  0.299  9 | **-0.717**  **0.030**  **9** | 0.441  0.235  9 | -0.280  0.466  9 | -0.252  0.548  8 | -0.481  0.190  9 | -0.237  0.539  9 | -0.009  0.982  9 | -0.170  0.662  9 |
| Avoidant  ρ =  *p* =  N = | -0.457  0.302  7 | -0.360  0.428  7 | -0.395  0.381  7 | -0.224  0.629  7 | -0.019  0.968  7 | -0.405  0.368  7 | -0.070  0.882  7 | 0.395  0.439  6 | 0.361  0.426  7 | -0.450  0.310  7 | **-0.927**  **0.003**  **7** | -0.574  0.178  7 |
| Attention deficit hyperactivity  ρ =  *p* =  N = | -0.193  0.619  9 | -0.287  0.454  9 | -0.176  0.650  9 | 0.292  0.446  9 | -0.287  0.454  9 | -0.365  0.334  9 | 0.028  0.944  9 | 0.327  0.429  8 | 0.295  0.440  9 | -0.167  0.668  9 | -0.638  0.064  9 | -0.492  0.179  9 |
| Antisocial  ρ =  *p* =  N = | 0.102  0.827  7 | 0.092  0.844  7 | -0.075  0.872  7 | 0.346  0.447  7 | -0.269  0.559  7 | -0.046  0.922  7 | -0.061  0.896  7 | 0.801  0.056  6 | 0.495  0.258  7 | -0.148  0.751  7 | -0.673  0.097  7 | **-0.857**  **0.014**  **7** |

Correlations between ASEBA questionnaires and QoL questionnaires for patients aged 16 and older (N=9). Bold and underlined results are significant correlations. For patients 16-18 years old (N=2), the ASEBA questionnaire did not calculated the scales intrusive, depressed, avoidant and antisocial. The domain sex could not be calculated for one of the patients due to missing answers in the questionnaire.
