# Supplementary material for: Emotional and behavioral problems, quality of life and metabolic control in NTBC-treated Tyrosinemia type 1 patients
Source: Orphanet J Rare Dis. 2019 Dec 4;14:285. doi: 10.1186/s13023-019-1259-2 (PMC6894144; doi:10.1186/s13023-019-1259-2)
Supplement: Supplementary file 3 — Additional file 3. Summarized results of correlation analyses between phenylalanine and tyrosine concentrations and neurocognitive outcome scores. Summarized results of correlation analyses between phenylalanine and tyrosine concentrations and neurocognitive outcome scores. Only scales with significant correlations, with p-values < 0.05, are shown. ρ = Spearman’s rho. For ASEBA, positive correlations indicate that higher levels are related to poorer outcomes, whereas negative correlations indicate that higher levels are related to better outcomes; for HR-QoL, positive correlations indicate that higher levels are related to better outcomes, whereas negative correlations indicate that higher levels are related to poorer outcomes. [file 13023_2019_1259_MOESM3_ESM.docx]

**Additional file 3**. Summarized results of correlation analyses between phenylalanine and tyrosine concentrations and neurocognitive outcome scores.

|  |  | **First year Phe** | **First year Tyr** |  | **Lifetime Phe** | **Lifetime Tyr** |  | **Last year Phe** | **Last year Tyr** |
| --- | --- | --- | --- | --- | --- | --- | --- | --- | --- |
| **ASEBA scales** | | |  |  |  |  |  |  |  |
| Withdrawn/  depressed |  |  |  |  |  |  |  | ρ = 0.411; *p* = 0.030 | ρ = 0.492; *p* = 0.008 |
| Somatic complaints |  | ρ = -0.421; *p* = 0.040 |  |  |  |  |  |  |  |
| Social problems |  | ρ = -0.457; *p* = 0.043 | ρ = -0.608; *p* = 0.004 |  |  |  |  |  |  |
| Thought problems |  | ρ = -0.430; *p* = 0.040 |  |  |  |  |  |  |  |
| Attention problems |  | ρ = -0.513; *p* = 0.010 | ρ = -0.598; *p* = 0.002 |  |  |  |  |  |  |
| Delinquent behavior |  |  | ρ = -0.417; *p* = 0.043 |  |  |  |  |  |  |
| Aggressive behavior |  |  | ρ = -0.438; *p* = 0.032 |  |  |  |  |  |  |
| **ASEBA DSM scales** | | |  |  |  |  |  |  |  |
| Affective problems |  |  | ρ = -0.449; *p* = 0.047 |  |  |  |  | ρ = 0.418; *p* = 0.047 |  |
| Attention deficit hyperactivity problems |  |  | ρ = -0.493; *p* = 0.014 |  |  |  |  |  |  |
| Anxiety problems |  |  |  |  |  |  |  | ρ = 0.381; *p* = 0.045 |  |
| Conduct problems |  |  | ρ = -0.667; *p* = 0.001 |  |  |  |  |  |  |
| **HR-QoL children** | | |  |  |  |  |  |  |  |
| Autonomy |  | ρ = 0.609; *p* = 0.016 |  |  | ρ = 0.517; *p* = 0.034 |  |  |  |  |
| Positive emotions |  |  |  |  |  |  |  |  | ρ = -0.505; *p* = 0.033 |
| **HR-QoL >16 years** | | |  |  |  |  |  |  |  |
| Cognition |  | ρ = -0.943; *p* = 0.005 |  |  | ρ = -0.829; *p* = 0.021 |  |  |  |  |
| Social contacts |  |  |  |  | ρ = -0.802; *p* = 0.030 |  |  | ρ = -0.802; *p* = 0.030 |  |

Summarized results of correlation analyses between phenylalanine and tyrosine concentrations and neurocognitive outcome scores. Only scales with significant correlations, with *p*-values <0.05, are shown. ρ = Spearman’s rho. For ASEBA, positive correlations indicate that higher levels are related to poorer outcomes, whereas negative correlations indicate that higher levels are related to better outcomes; for HR-QoL, positive correlations indicate that higher levels are related to better outcomes, whereas negative correlations indicate that higher levels are related to poorer outcomes.
